# Short-term visual deprivation reduces interference effects of task-irrelevant facial expressions on affective prosody judgments

**DOI:** 10.3389/fnint.2015.00031

**Published:** 2015-04-22

**Authors:** Ineke Fengler, Elena Nava, Brigitte Röder

**Affiliations:** ^1^Biological Psychology and Neuropsychology, Faculty of Psychology and Human Movement Science, Institute for Psychology, University of HamburgHamburg, Germany; ^2^Department of Psychology, University of Milan-BicoccaMilan, Italy; ^3^NeuroMI Milan Center for NeuroscienceMilan, Italy

**Keywords:** neuroplasticity, short-term visual deprivation, prosodic discrimination, emotions, multisensory

## Abstract

Several studies have suggested that neuroplasticity can be triggered by short-term visual deprivation in healthy adults. Specifically, these studies have provided evidence that visual deprivation reversibly affects basic perceptual abilities. The present study investigated the long-lasting effects of short-term visual deprivation on emotion perception. To this aim, we visually deprived a group of young healthy adults, age-matched with a group of non-deprived controls, for 3 h and tested them before and after visual deprivation (i.e., after 8 h on average and at 4 week follow-up) on an audio–visual (i.e., faces and voices) emotion discrimination task. To observe changes at the level of basic perceptual skills, we additionally employed a simple audio–visual (i.e., tone bursts and light flashes) discrimination task and two unimodal (one auditory and one visual) perceptual threshold measures. During the 3 h period, both groups performed a series of auditory tasks. To exclude the possibility that changes in emotion discrimination may emerge as a consequence of the exposure to auditory stimulation during the 3 h stay in the dark, we visually deprived an additional group of age-matched participants who concurrently performed unrelated (i.e., tactile) tasks to the later tested abilities. The two visually deprived groups showed enhanced affective prosodic discrimination abilities in the context of incongruent facial expressions following the period of visual deprivation; this effect was partially maintained until follow-up. By contrast, no changes were observed in affective facial expression discrimination and in the basic perception tasks in any group. These findings suggest that short-term visual deprivation per se triggers a reweighting of visual and auditory emotional cues, which seems to possibly prevail for longer durations.

## Introduction

Neuroplasticity refers to the fundamental property of the brain to change its structural and functional characteristics to better suit environmental demands (Kolb, 1998; Shaw and McEachern, 2001; Lövdén et al., 2010). Although neuroplasticity has been commonly observed during early development (e.g., Bavelier and Neville, 2002; Knudsen, 2004; Lewkowicz and Röder, 2012), growing literature has reported striking changes in the adult brain too (Lövdén et al., 2010; May, 2011; Lövdén et al., 2013), suggesting that the mature brain keeps a considerable adaptivity. Furthermore, research on individuals with sensory deficits (i.e., blind and deaf individuals) has provided evidence that once a sensory modality is lost, the remaining modalities reorganize (Bavelier and Neville, 2002; Merabet and Pascual-Leone, 2010; Pavani and Röder, 2012). Cross-modal plasticity has been observed irrespective of the age at deprivation onset and seems to be predominantly induced by sensory deprivation *per se* and not by the extended use of the spared modalities (e.g., Neville and Lawson, 1987; Bavelier et al., 2001; Fine et al., 2005).

More recent studies have employed novel approaches to study experience dependent changes in the large-scale organization of the brain (Bullmore and Sporns, 2009). For example, it has been demonstrated that cross-modal plasticity likely involves changes in functional (Klinge et al., 2010; Schepers et al., 2012) and structural connectivity (Voss and Zatorre, 2015).

Recently, several studies have investigated the effects of reversible total visual deprivation in healthy humans – as achieved by blindfolding participants – on the remaining sensory (i.e., tactile and auditory) functions (Pascual-Leone and Hamilton, 2001; Kauffman et al., 2002; Pollok et al., 2005; Merabet et al., 2008). Before and after visual deprivation participants were tested on behavioral tasks involving the non-deprived modalities. Results showed enhanced performance in the tested non-deprived modalities (Kauffman et al., 2002; Merabet et al., 2008). In addition, neuroimaging data were acquired that pointed to cross-modal plasticity related to the visual deprivation (Pascual-Leone and Hamilton, 2001; Pollok et al., 2005; Merabet et al., 2008).

It should be noted that the blindfolded participants in these studies underwent some (implicit) training in the tested modality, thus not providing conclusive results as to the independent effects of sensory deprivation and the more extensive use of the non-deprived modalities, respectively. For example, Kauffman et al. (2002) tested 24 sighted individuals randomly assigned to one of four groups: blindfolded and stimulated (group 1), blindfolded and non-stimulated (group 2), non-blindfolded and stimulated (group 3), and non-blindfolded and non-stimulated (group 4). For 5 days, blindfolded participants were visually deprived, and stimulated participants underwent daily Braille reading lessons (4 h each; using their right index finger) as well as tactile game training (e.g., practicing tactile dominos; 2 h each; involving predominantly the right index finger too). The ability to recognize Braille characters was tested on day 1, day 3, and day 5 in all participants; groups 3 and 4 were blindfolded during the testing sessions. Results showed that during the course of the study, both groups 1 and 2 improved their Braille discrimination ability significantly more than groups 3 and 4 (this effect was specific for the right index finger). However, it has to be noted that participants of group 2, though not explicitly engaged in a tactile training, implicitly had to use touch more extensively, e.g., for daily activities, as well. Therefore, it is not clear yet whether practice or blindfolding caused the advantages in the blindfolded groups.

Interestingly, several later studies have shown that enhanced tactile and auditory functions emerge even after short-term visual deprivation of a few hours. For example, Facchini and Aglioti (2003) assessed the performance of two groups of participants in a tactile grating orientation task (GOT) before and after a time interval of 90 min. Both groups were blindfolded during testing, but the experimental group remained blindfolded between the testing sessions. Only the experimental group showed lower thresholds in the GOT task in the second as compared to the first session.

Similarly, Lewald (2007) assessed sound localization accuracy before and after 110 min of visual deprivation in two groups. All participants repeatedly performed a task in which they had to point to acoustic targets while being blindfolded. During a 110 min break between the first and the second session, the experimental group remained blindfolded. In contrast to the control group, this group showed a reduced mean constant error of sound localization in the second session. However, Lewald (2007) did not control for auditory training effects resulting from auditory input during the break in which the experimenter usually engaged participants in a conversation. Therefore, the observed performance improvement in the experimental group cannot be unequivocally attributed to the blindfolding alone. The same critique applies to a study by Landry et al. (2013), who investigated the effects of a 90 min period of blindfolding on a harmonicity discrimination task administered before and after this period. In this study, participants were asked to decide whether the third tone in a series of six pure tones was in tune with the other five. During task completion (about 5 min), all participants were blindfolded. The authors found that only the experimental group significantly improved their harmonicity discrimination ability from session 1 to session 2. However, all participants were asked to (watch and) listen to a movie during the 90 min inter-session interval, which may have influenced their performance.

The effects of visual deprivation have consistently been shown to be reversible, meaning that performance returns to the level observed prior to visual deprivation. For example, the tactile and auditory enhancement found in the studies of Facchini and Aglioti (2003) and Lewald (2007) had returned to baseline after 180 and 130 min of re-exposure to light, respectively. Landry et al. (2013) reported a progressive return to baseline within a time period of only 60 min after blindfold release. The effects of long-term visual deprivation seem to be longer lasting, but have been found to be lost after 12–24 h as well (Pascual-Leone and Hamilton, 2001; Merabet et al., 2008). These observations are compatible with the findings of Boroojerdi et al. (2000), who visually deprived participants for 180 min to measure changes in the excitability of the visual cortex during and after that period. The authors showed by applying transcranial magnetic stimulation (TMS) on the occipital cortex that phosphenes (i.e., illusionary light flashes) were elicited with a lower threshold following 45 min of visual deprivation and that this effect persisted for 120 min after deprivation offset, after which phosphene induction returned to the level prior to deprivation (see Pitskel et al., 2007, for similar results following 5 days of visual deprivation). Boroojerdi et al. (2000) additionally showed an increased activity of the visual cortex in response to photic stimulation within the period of 60 min after visual deprivation onset and 30 min after re-exposure to light (as measured with fMRI).

In sum, a number of studies have provided evidence that blindfolding healthy adults can trigger functional changes even in a short period, demonstrating remarkably plasticity of the human brain. However, these studies have left some questions unsolved. First, the tasks used so far have focused on basic unisensory perception, thus leaving unanswered the question of whether visual deprivation can influence multisensory perceptual processing too. Second, the specific effects of visual deprivation on the one hand and stimulation of the non-deprived sensory modalities on the other hand are yet not clear. Finally, these studies did not investigate possible long-term effects.

In the present study, we addressed these issues by longitudinally testing (i.e., on three sessions) three age-matched groups of young healthy adult participants on an audio*–*visual emotion discrimination task. To observe whether visual deprivation affects basic multisensory and unisensory perception, we additionally tested our participants on the so-called “Colavita effect” (e.g., Colavita, 1974), and on two unisensory perceptual threshold measures. After approximately 4 weeks from the first testing session, one group of participants stayed in a completely dark room for a period of 3 h, while a control group was exposed to normal light. During this period, both groups performed a set of auditory tasks. To disambiguate the role of visual deprivation *per se* and influences from intense auditory stimulation on auditory, visual, and audio–visual processing, we visually deprived an additional group of participants who concurrently performed unrelated (i.e., tactile) tasks. Finally, to examine short-term as well as long-term effects, all groups were tested shortly after (*M* = 8 h) the 3 h intervention period and at a 4 weeks follow-up.

We hypothesized that if visual deprivation *per se* were responsible for changes underlying improved performance in tasks not involving the deprived modality, we would observe improvements in the two visually deprived groups, but not in the non-deprived (ND) group. By contrast, if both perceptual training and deprivation had independent or mutually enhancing effects, we hypothesized to observe higher performance in the group who performed auditory tasks during the visual deprivation period.

## Materials and Methods

### Participants

Forty-seven adult students, all recruited from the University of Hamburg, took part in the study. Participants were randomly assigned to one of three groups: a visually deprived group performing auditory tasks during deprivation (VD1): *n* = 17, female: 12, left-handed: 2, mean age: 23 years, age range: 19–32 years; a non-visually deprived group (ND) performing the same auditory tasks as VD1: *n* = 15, female: 10, left-handed: 2, mean age: 24 years, age range: 19–44 years; a visually deprived group performing tactile tasks during visual deprivation (VD2): *n* = 15, female: 10, left-handed: 2, mean age: 25 years, age range: 20–38 years. All participants had no history of neurological disease and reported normal hearing and normal or corrected-to-normal vision. All participants gave informed consent prior to participation and received either course credits or a monetary compensation (7€/hour). Participants who were assigned to the visually deprived groups were specifically informed about the type of procedure they would undergo (see General Procedure). All participants gave informed consent prior to participation. The study was approved by the ethical board of the German Psychological Society.

### Description of Experiments

#### Emotion Discrimination

The stimuli were adapted from the study of Föcker et al. (2011; see there for a detailed description). In short, visual stimuli consisted of short video streams with faces mouthing bisyllabic German pseudowords (“lolo,” “tete,” or “gigi”) presented on a computer screen (viewing distance: 60 cm). Auditory stimuli consisted of voices speaking out the same bisyllabic pseudowords pronounced by the faces in the videos (the level of the sound tracks varied between 65 and 72 dB). The faces and the voices expressed one of four emotions when mouthing/speaking out the syllables (Happy, sad, angry, or neutral). Responses were registered using a keyboard. Written instructions were provided. Participants were instructed to respond as quickly and as accurately as possible using their dominant hand. Stimulus presentation and response recording was effected via the Presentation program by Neurobehavioral Systems.

Unimodal visual and auditory stimuli were face and voice presentations only, respectively. Audio–visual congruent trials presented face–voice pairings with face and voice displaying the same emotion and audio–visual incongruent trials presented face–voice pairings with face and voice displaying a different emotion. Note that, for both congruent and incongruent audio–visual trials the audio track and the video stream originated from independent recordings. This was done to compensate for possible minimal temporal misalignments when independent audio and visual streams were combined (see Föcker et al., 2011, for details).

Each trial began with a 500 ms audio–visual warning signal (a gray circle of two of visual angle, combined with multi-talker babble noise) to orient the participants’ attention to the stimuli (same procedure as in Föcker et al., 2011; a bimodal rather than a unimodal warning signal was used to avoid a modality priming effect). Then, after a variable inter-stimulus interval (ISI; 600–700 ms, uniform distribution), participants were presented with a face alone (i.e., a video stream), a voice alone (i.e., an audio track), or a face–voice pairing (i.e., video and audio stream). In one experimental block, the participants had to categorize the emotion and rate the intensity of the emotional expression of the faces (“attend faces”). In another block, they had to categorize the emotion and rate the intensity of the emotional expression of the voices (“attend voices”). While both blocks enclosed audio–visual congruent and audio–visual incongruent trials, the “attend faces” block additionally comprised unimodal visual trials and the “attend voices” block additionally enclosed unimodal auditory trials.

Each stimulus was presented twice, with an ISI of 3 s. Following the first presentation, participants categorized the displayed emotion by pressing one of four adjacent marked buttons on the keyboard. After the second presentation, participants rated the intensity of the emotional expression. To this end, a visual analog scale titled was presented on the screen (50 ms after stimulus offset) that ranged from 1 (*low*) to 5 (*high*) and the participants typed in one of the corresponding numbers on the keyboard. Subsequently, the next trial was presented. Up to 10 practice trials were run to familiarize the participants with the task. The experiment took, on average, 50 min to complete.

#### Colavita Visual Dominance Effect

The stimuli were adapted from Nava and Pavani (2013). Stimuli consisted of 50 ms tone bursts (4 KHz tone at 65 dB) emitted simultaneously from two loudspeakers and 50 ms light flashes from a red light-emitting diode (LED). Participants sat at approximately 60 cm from the LED and loudspeakers. The loudspeakers were situated next to each other and covered by a black cloth that did not shield the sounds. The LED was centrally attached on top of the cloth (height: 20 cm), facing the participant. Responses were registered via a response device with three horizontally aligned buttons. Written instructions were provided. Participants were instructed to respond as quickly and as accurately as possible using their dominant hand. Stimulus programming and presentation as well as response recording was effected via the Presentation program.

On each trial, participants were either presented with an auditory stimulus, a visual stimulus, or an audio–visual stimulus (i.e., tone burst and light flash presented simultaneously). Eight experimental blocks with a random sequence of 40 auditory, 40 visual, and 30 audio–visual stimuli each were administered. The participants were instructed to press the left button of the response device when perceiving an auditory stimulus, the middle button when perceiving a visual stimulus, and the right button when perceiving an audio–visual stimulus. They were given 1700 ms to provide a response; after an additional inter-trial interval (ITI) of 500 ms, the next trial was presented. The experiment took, on average, 40 min to complete.

#### Auditory Detection and Discrimination Thresholds

Experiments used a classical adaptive procedure (Green, 1990, 1993) implemented in a freely downloadable Matlab toolbox (Grassi and Soranzo, 2009). In particular, we tested the participants on a pitch change detection and pitch discrimination task. Responses were registered using a keyboard. Written instructions were provided for each task. Participants were instructed to respond as accurately as possible using their dominant hand.

In the pitch change detection task, participants heard five tones consecutively played for 100 ms each, with ISIs of 150 ms (at 65 dB). The employed tones were complex tones with three harmonics, gated on and off with two 10-ms raised cosine ramps. The first three as well as the fifth tone always had a frequency of 300 Hz; the frequency of the fourth tone was varied adaptively to find the participant’s threshold for pitch change detection. On 96 trials (three blocks of 32 trials each), participants had to indicate whether or not the pitch of the fourth tone was different from the pitch of the other four tones by typing in a 1 or a 0, respectively. After a confirmation via enter and an (ITI) of 150 ms, the next trial was presented. No feedback was given.

In the pitch discrimination task, three tones were consecutively played for 250 ms each (at 65 dB), separated by ISIs of 150 ms. The employed tones were complex tones with four harmonics, gated on and off with two 10-ms raised cosine ramps. While two of the tones always had a frequency of 330 Hz, one tone’s frequency was varied adaptively to find the participant’s threshold for pitch discrimination. On 140 trials (five blocks of 28 trials each), participants had to indicate whether the first, second, or third tone was different in pitch as compared to the other two by typing in a 1, 2, or 3, respectively. After a confirmation via enter and an ITI of 150 ms, the next trial was presented. No feedback was given.

The experiment took, on average, 15 min to complete.

#### Visual Discrimination Thresholds

Visual stimuli were presented on an analogue oscilloscope (TRIO, model CS-1577) placed centrally in front of the participant. The viewing distance was kept at 100 cm. The set-up of the oscilloscope was adapted to the participant’s height so that the monitor was at eye level for every individual. Responses were registered using a response device with two buttons which was held by the participant. In two different tasks, the stimuli were aligned horizontally and vertically, respectively. Written instructions were provided for each task. Participants were instructed to respond as accurately as possible using their dominant hand.

On every trial, two parallel lines [length: ca. 53.11 min of arc (MOA), width: 5.90 MOA] were displayed centrally on the oscilloscope’s 8.3 cm × 10.3 cm monitor for 50 ms, either horizontally (horizontal task) or vertically (vertical task). On the first trial, the lines were separated by ca. 48.94 MOA. The line separation of the following trials varied adaptively to find the participant’s threshold for visual discrimination [controlled by a Presentation script implementing parameter estimation by sequential testing (PEST) algorithm, see Taylor and Creelman, 1967]. On each trial of the horizontal task, participants had to indicate whether the left line was lower or higher as compared to the right line by pressing the left or the right button, respectively. On each trial of the vertical task, participants had to indicate whether the upper line was left or right as compared to the lower line by pressing the left or the right button, respectively. Immediately after a response was provided, the next trial was presented. No feedback was given. The experiment took, on average, 10 min to complete.

### General Procedure

**FIGURE 1:**
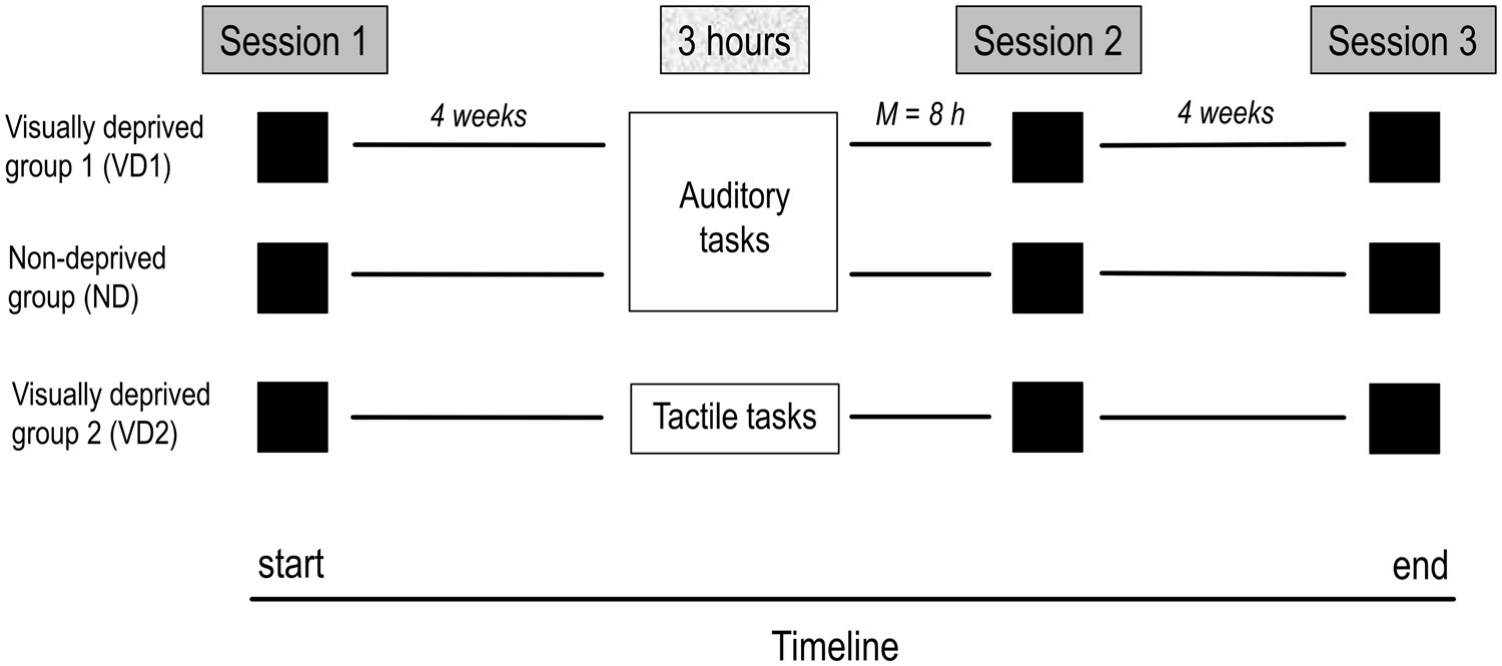
**Timeline of the study.** The study comprised three testing sessions, separated by 4 weeks on average, and a 3 h period of visual deprivation and/or administration of a task battery (VD1: visual deprivation and auditory tasks, ND: auditory tasks, VD2: visual deprivation and tactile tasks) preceding the second testing session by 8 h on average. On each session, all groups were tested on the same tests, including the audio–visual emotion discrimination task, the Colavita effect, and visual and auditory threshold measures.

**Figure 1** displays the timeline of the study. All participants took part in three identical testing sessions including the four experiments. Approximately 4 weeks after the first testing session, VD1 and VD2 were visually deprived for 3 h while performing auditory and tactile tasks, respectively, ND performed the same set of auditory tasks as VD1 but remained non-deprived. The second testing session shortly followed the 3 h period (i.e., same or next day) and the third testing session took place at 4 week follow-up. The order of experiments administered in the testing sessions was counterbalanced across participants. All experiments were run in a normally lit room of the Biological Psychology and Neuropsychology Laboratory at the University of Hamburg.

Visual deprivation was achieved by keeping the participants in a completely dark room for 3 h. Whereas the VD1 participants were visually deprived in a room made available by the association Dialogue in the Dark^1^, the VD2 participants were visually deprived in a room of the Biological Psychology and Neuropsychology Laboratory at the University of Hamburg, which was sound-shielded as well. In either room, furniture and task equipment were hazard-free. Before entering the dark rooms, the VD1 and VD2 participants were informed about safety procedures in case in emergency (i.e., alarm bell, emergency light switches).

The auditory tasks that were administered to the VD1 and ND^2^ participants particularly focused on improving communication and emotion understanding in healthy participants (e.g., by role playing). The VD2 participants, on the contrary, were tested on a battery of tactile tasks, particularly investigating mental rotation skills (e.g., on a custom-made tactile version of the mirror images task from the Wilde Intelligence Test; Kersting et al., 2008; see **Table 1** for an overview of the auditory and tactile tasks). Note that the auditory tasks were performed in small groups (five participants) whereas the tactile tasks were performed individually to avoid auditory stimulation through interpersonal interactions. While performing the tactile tasks, VD2 participants additionally wore ear plugs to attenuate any sounds elicited by the manipulation of the response items (i.e., attaching and detaching items via hook-and-pile).

**Table 1:** Overview of the tasks administered to the participants during the 3 h time period preceding the second testing session.

Auditory tasks (Administered to VD1 and ND participants in groups of five)	Tactile tasks (Administered to VD2 participants individually)
Description task: Description of different persons based on non-visual aspects only Prosody production task 1: Repeated articulation of the words “yes” and “no” using different prosodic variations (e.g., happy, sad, angry, etc.) Prosody production task 2: Presentation of short scenes/dialogues with various affective prosody Prosody understanding task: Repeated evaluation of prosody acted out by the trainer	Mirror images task^a^: Discriminating mirror images and non-mirror images on 20 rows of five objects each Copying task 1: Reproduction of 11 rows of five to eight Landolt rings with different orientations each Copying task 2: Reproduction of nine rows of eight 3-dot objects with different orientations each Counting task: Counting the number of times each item included in the copying tasks was to be found on the templates

Materials of the tactile tasks were custom-made; templates were embossed plastic sheets, and response items were made of foam rubber. ^a^Adapted from the Wilde Intelligenz Test (WIT-2; Kersting et al., 2008).

The auditory and the tactile task battery took about 3 h to complete, respectively. Visual deprivation was terminated irrespective of task completion after 3 h were up.

Note that data analyses are described together with the results.

## Results

With respect to the Emotion discrimination experiment and the Colavita visual dominance effect, we included 11 participants in each group. Fourteen additional participants were excluded from analyses because they did not attend all sessions. With respect to the auditory and visual threshold measures, we included eight participants in each group. Twenty-three participants were discarded from final analyses because they did not attend all sessions (*n* = 14) or because of extreme outliers (*n* = 9).

The visual deprivation period was tolerated by all participants. No hallucinations or other discomforts were reported during or after this period.

For clarity, below we describe the results of our study separately for each experiment. Because the data were not normally distributed (as assessed by Shapiro–Wilk tests with *p* < 0.05) and homogeneity of variances across the within-subject factors was not given for any group (as assessed by Levene’s tests, all *p* < 0.05), we used non-parametric tests. For each experiment and each group, we computed permutation based Friedman rank sum tests with Monte Carlo point estimates of the exact *p*-values (9999 Monte Carlo replications; using the R program, package *coin*, version 1.0–23) to test for inter-session differences. For *post hoc* comparisons, we used exact signed rank Wilcoxon tests (using the R program, package *coin*, version 1.0–23 as well; zeros handled according to Wilcoxon; *p*-values adjusted using the Holm–Bonferroni method of sequential correction).

### Emotion Discrimination

We compared accuracy and intensity ratings between sessions, separately for experimental blocks (“attend faces,” “attend voices”) and conditions. Note that in the following paragraph, we focus on the results of those Friedman rank sum tests that yielded significant results; please see **Table 2** for details regarding the non-significant Friedman rank sum tests.

**Table 2:** Overview of the significant and the non-significant results of the permutation based Friedman rank sum tests computed on the accuracy and intensity rating data from the emotion discrimination experiment, separately for each group, block, and condition.

			Accuracy		Intensity rating	
Group	Block	Condition	$\chi_{r}^{2}$	*p*-value	$\chi_{r}^{2}$	*p*-value
VD1^a^	‘Attend faces’	Unimodal	2.58	0.30	0.71	0.75
		Congruent	0.39	0.87	0.16	0.95
		Incongruent	0.14	0.96	1.00	0.65
	‘Attend voices’	Unimodal	4.16	0.13	1.19	0.59
		Congruent	1.17	0.58	0.67	0.77
		Incongruent	6.95	0.03	0.84	0.69
ND^b^	‘Attend faces’	Unimodal	7.78	0.02	1.6	0.47
		Congruent	1.28	0.55	3.05	0.22
		Incongruent	4.33	0.13	3.17	0.22
	‘Attend voices’	Unimodal	4.10	0.13	0.4	0.87
		Congruent	0.21	0.91	1.95	0.41
		Incongruent	1.55	0.48	0.72	0.74
VD2^c^	‘Attend faces’	Unimodal	3.8	0.18	3.50	0.18
		Congruent	3.95	0.14	4.56	0.11
		Incongruent	0.88	0.70	5.6	0.06
	‘Attend voices’	Unimodal	4.10	0.13	1.14	0.59
		Congruent	0.21	0.92	0.42	0.87
		Incongruent	6.49	0.03	2.72	0.28

Statistics concern the difference between all three testing sessions. ^a^VD1: Visually deprived group performing auditory tasks; ^b^ND: Non-deprived group performing auditory tasks; ^c^VD2: Visually deprived group performing tactile tasks (*n* = 11 for all groups).

#### Accuracy

**FIGURE 2:**
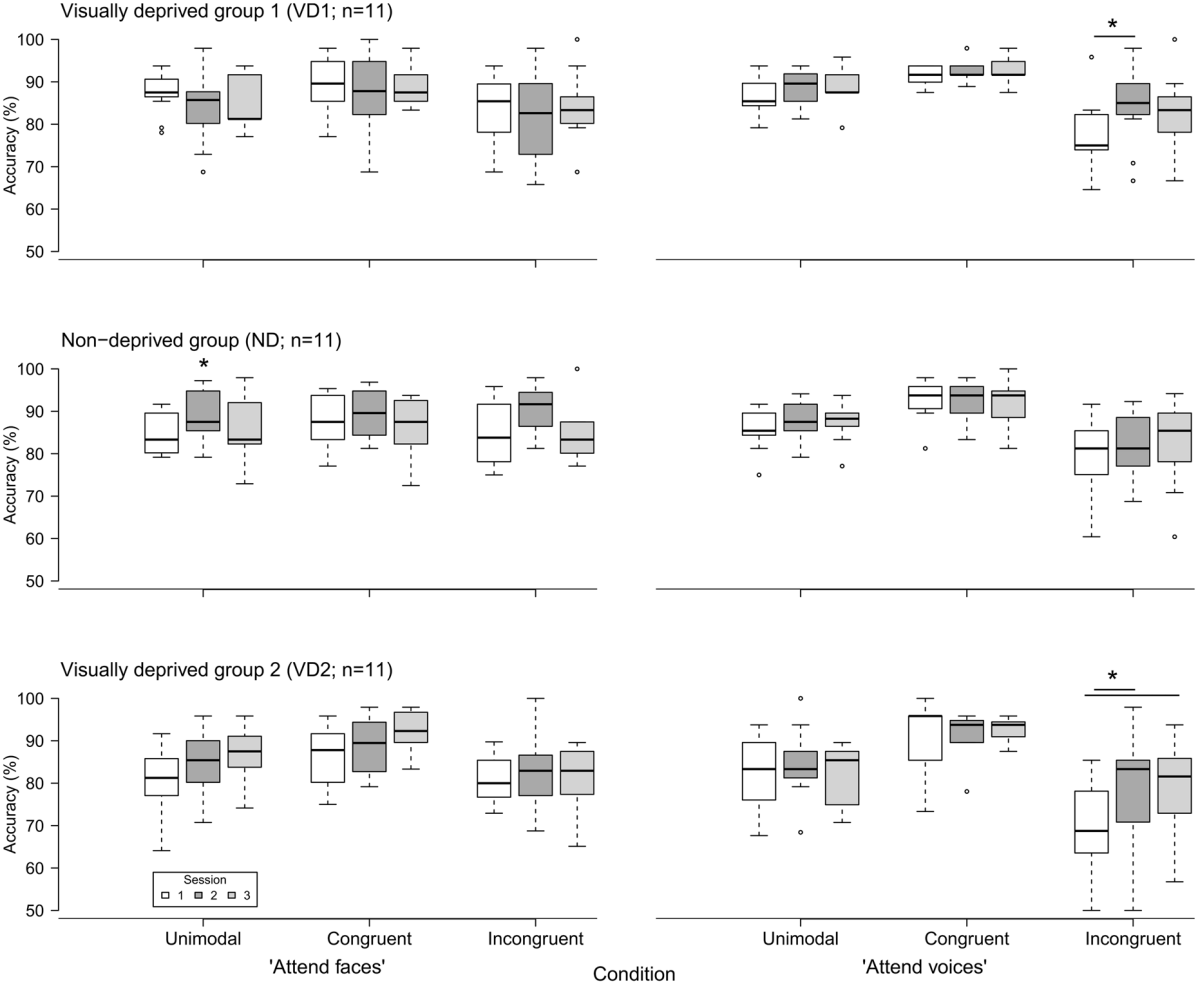
**Accuracy of emotion discrimination (in percent) in the three groups (VD1, ND, VD2; *n* = 11 each) as a function of session (1, 2, and 3), block (“attend faces,” “attend voices”), and condition (unimodal, congruent, incongruent).** Whiskers denote the lowest and highest data points within ±1.5*interquartile range. Note that the y axis starts at 50%. Significant inter-session differences as discovered by *post hoc* tests are highlighted by asterisks and lines between the medians. The single asterisk indicates a main effect of session without significant *post hoc* tests.

**Figure 2** displays the accuracy of response in the “attend faces” and “attend voices” blocks between sessions, separately for groups.

VD1 showed a significant difference for the incongruent condition of the “attend voices” block ($\chi_{r}^{2}$ = 6.95, *p* = 0.03). *Post hoc* comparisons revealed that this difference was caused by VD1 improving in prosodic discrimination accuracy in the incongruent condition following visual deprivation, namely between session 1 and session 2 (session 1: *Mdn* = 75.00%; *range* = 64.58–95.83%, session 2: *Mdn* = 85.00%; *range* = 66.67–97.92%; *Z* = -2.51, *p* = 0.01, one-tailed exact signed rank Wilcoxon test, *p* values adjusted by Holm–Bonferroni correction). The Holm–Bonferroni corrected *p*-value of the *post hoc* comparison between session 1 and session 3 just failed to reach the level of significance (session 3: *M* = 83.33%; *range* = 66.67–100%; *Z* = -1.74, *p* = 0.09). This result at least points toward a maintenance of the intervention effect. There was no significant difference between session 2 and session 3 (*Z* = 0.99, *p* = 0.84). Similar results were obtained for group VD2, which showed a significant difference in the incongruent condition of the “attend voices” block too ($\chi_{r}^{2}$ = 6.49, *p* = 0.03). *Post hoc* comparisons revealed that this difference was caused by better discrimination of prosody in the incongruent condition in session 2 (*Mdn* = 83.33%; *range* = 50.00–97.92%; *Z* = -2.20, *p* = 0.03) and session 3 (*Mdn* = 81.58%; *range* = 56.76–93.75%; *Z* = -2.49, *p* = 0.01, one-tailed exact signed rank Wilcoxon tests, *p*-values adjusted by Holm–Bonferroni correction) in comparison to session 1 (*Mdn* = 68.75%; *range* = 50.00–85.42%) as well. There was no significant difference between session 2 and session 3 (*Z* = -0.66, *p* = 0.28).

In contrast, ND showed a significant difference in the unimodal condition of the “attend faces” block ($\chi_{r}^{2}$ = 7.78, *p* = 0.02). *Post hoc* comparisons did not reveal significant differences between any two sessions, however, [session 1 (*Mdn* = 83.33%; *range* = 79.17–91.67%) vs. session 2 (*Mdn* = 87.50%; *range* = 79.17–97.22%): *Z* = -1.78, *p* = 0.25; session 1 vs. session 3 (*Mdn* = 83.33%; *range* = 72.92–97.92%): *Z* = -0.65, *p* = 0.57; session 2 vs. session 3: *Z* = 1.72, *p* = 0.25, two-tailed exact signed rank Wilcoxon tests, *p*-values adjusted by Holm–Bonferroni correction].

#### Intensity Rating

Emotion intensity ratings did not vary across sessions for any group (all *p* > 0.05, permutation based Friedman rank sum tests).

### Colavita Visual Dominance Effect

For all participants and in all sessions and conditions (auditory, visual, audio–visual), misses (i.e., late or no responses) were negligible (i.e., below 2%). Overall, the percentage of errors (i.e., incorrect responses) was 3.62% in the first session, 3.37% in the second session, and 3.55% in the third session. This percentage did not differ between sessions ($\chi_{r}^{2}$ = 0.15, *p* = 0.94, permutation based Friedman rank sum test). See **Table 3** for an overview of the misses and correct responses in each condition, separately for each group and each session.

**Table 3:** Percentage of correct responses and misses for each condition as well as percentage of visual and auditory responses in erroneous audio–visual trials of the audio–visual discrimination task probing the Colavita effect, separately for each group and each session.

		Session 1			Session 2			Session 3		
		AV	V	A	AV	V	A	AV	V	A
VD1^a^	Correct responses	93.30 (1.67–15.42)	97.81 (0.00–7.64)	98.44 (0.31–8.18)	97.08 (0.00–10.42)	97.19 (0.63–11.25)	98.75 (0.00–4.72)	97.50 (0.42–11.25)	97.19 (0.31–9.06)	98.13 (0.00–5.97)
	Misses	0.00 (0.00–1.42)	0.00 (0.00–1.88)	0.06 (0.00–0.63)	0.00 (0.00–0.00)	0.00 (0.00–0.63)	0.00 (0.00–0.63)	0.00 (0.00–1.25)	0.00 (0.00–1.56)	0.00 (0.00–0.63)
	Erroneous responses to AV	V: 3.35 (1.67-8.33)			V: 2.08 (0.00-9.17)			V: 0.83 (0.42-10.83)		
		A: 1.25 (0.00-7.50)			A: 0.83 (0.00-3.75)			A: 0.94 (0.00-2.95)		

ND^b^	Correct responses	92.92 (0.83–13.75)	96.88 (0.00–7.81)	98.44 (0.00–4.69)	95.00 (0.24–12.08)	96.25 (0.63–8.13)	98.44 (0.00–5.00)	95.00 (0.42–16.67)	98.13 (0.31–7.50)	97.50 (0.31–5.94)
	Misses	0.00 (0.00–0.00)	0.00 (0.00–0.31)	0.00 (0.00–0.31)	0.00 (0.00–1.00)	0.00 (0.00–0.00)	0.00 (0.00–0.00)	0.00 (0.00–0.00)	0.00 (0.00–0.00)	0.00 (0.00–0.00)
	Erroneous responses to AV	V: 4.67 (0.00–10.83)			V: 3.75 (0.42–7.50)			V: 4.17 (0.00–8.75)		
		A: 1.25 (0.00–2.92)			A: 1.25 (0.42–4.58)			A: 0.94 (0.00–8.33)		

VD2^c^	Correct responses	96.25 (0.83–11.25)	97.19 (0.31–7.19)	98.21 (0.31–6.41)	93.28 (0.42–10.00)	97.19 (0.94–8.44)	98.13 (0.63–6.25)	95.00 (0.83–11.67)	96.56 (1.88–7.81)	97.19 (0.31–4.06)
	Misses	0.00 (0.00–0.00)	0.00 (0.00–0.00)	0.00 (0.00–0.31)	0.00 (0.00–0.00)	0.00 (0.00–0.00)	0.00 (0.00–0.00)	0.00 (0.00–0.42)	0.00 (0.00–0.00)	0.00 (0.00–0.31)
	Erroneous responses to AV	V: 2.50 (0.42–8.33)			V: 2.52 (0.42–7.50)			V: 3.33 (0.83–6.67)		
		A: 1.25 (0.42–5.00)			A: 1.67 (0.00–4.20)			A: 1.25 (0.00–5.00)		

Displayed are medians with ranges in parentheses, all values in percent. ^a^VD1: Visually deprived group performing auditory tasks; ^b^ND: Non-deprived group performing auditory tasks; ^c^VD2: Visually deprived group performing tactile tasks (*n* = 11 for all groups); AV, Audio–visual condition; V, Visual condition/response; A, Auditory condition/response.

To investigate the presence of the Colavita effect, we calculated for each participant the distribution of visual and auditory responses in erroneous audio–visual trials. A higher number of visual responses indicate a Colavita effect. VD2 and ND showed a Colavita effect in all sessions (all *p* < 0.05, one-tailed exact signed rank Wilcoxon tests). VD1 showed a Colavita effect in sessions 1 and 2 (*p* < 0.05, one-tailed exact signed rank Wilcoxon tests), and a trend in session 3 (*Z* = 1.38, *p* = 0.10). **Table 3** provides an overview of the visual and auditory responses in audio–visual trials in each group and for each session.

Regarding the unimodal conditions, the percentage of errors was generally numerically larger in the visual as compared to the auditory condition (only for ND in session 3, the amount of auditory errors was larger). Significantly higher error rates in the visual as compared to the auditory condition were found for VD1 in session 3 (V: *Mdn* = 2.81%; range = 0.31–9.06%, A: *Mdn* = 1.88%; range = 0.00–5.97%; *Z* = -2.67, *p* < 0.01), for ND in session 2 (V: *Mdn* = 3.75%; range = 0.63–8.13%, A: *Mdn* = 1.56%; range = 0.00–5.00%; *Z* = -2.51, *p* < 0.01), and for VD2 in session 3 (V: *Mdn* = 3.44%; range = 1.88–7.81%, A: *Mdn* = 2.81%; range = 0.31–4.06%; *Z* = -2.22, *p* = 0.02).

For every participant, we calculated the difference between visual and auditory responses in audio–visual trials. This difference did not vary across sessions for any group (VD1: $\chi_{r}^{2}$ = 2.18, *p* = 0.36; ND: $\chi_{r}^{2}$ = 0.62, *p* = 0.78; VD2: $\chi_{r}^{2}$ = 2.36, *p* = 0.35, permutation based Friedman rank sum tests).

### Auditory Detection and Discrimination Thresholds

The detection threshold did not vary across sessions for any group (VD1: $\chi_{r}^{2}$ = 0.06, *p* = 0.99; ND: $\chi_{r}^{2}$ = 1.75, *p* = 0.53; VD2: $\chi_{r}^{2}$ = 4.75, *p* = 0.12, permutation based Friedman rank sum tests).

With respect to the discrimination threshold, we observed differences across sessions in ND ($\chi_{r}^{2}$ = 9.25, *p* < 0.01) and VD2 ($\chi_{r}^{2}$ = 7.75, *p* = 0.02), but no difference in VD1 ($\chi_{r}^{2}$ = 0.25, *p* = 0.97).

*Post hoc* comparisons revealed that ND was better at discriminating pitch in session 2 (*Mdn* = 2.10 Hz; *range* = 1.10–2.78 Hz; *Z* = 2.52, *p* = 0.02, two-tailed exact signed rank Wilcoxon test, *p* values adjusted by Holm–Bonferroni correction) as compared to session 1 (*Mdn* = 2.55 Hz; *range* = 1.21–5.59 Hz). There were no differences between session 3 (*Mdn* = 1.85 Hz; *range* = 0.76–4.93 Hz; *Z* = 1.68, *p* = 0.22) and session 1, and between session 2 and session 3 (*Z* = -0.42, *p* = 0.74). VD2 was better at discriminating pitch in session 3 (*Mdn* = 1.60 Hz; *range* = 0.87–2.66 Hz; *Z* = 2.52, *p* = 0.02) as compared to session 1 (*Mdn* = 2.57 Hz; *range* = 2.33–3.94 Hz). There were no differences between session 2 (*Mdn* = 1.99 Hz; *range* = 1.18–3.28 Hz; *Z* = 1.82, *p* = 0.16) and session 1, and between session 2 and session 3 (*Z* = 0.84, *p* = 0.46).

### Visual Discrimination Thresholds

For every participant, we calculated one mean threshold per session by averaging the horizontal and vertical threshold, respectively. The mean threshold did not vary across sessions for any group (VD1: $\chi_{r}^{2}$ = 0.47, *p* = 0.83; ND: $\chi_{r}^{2}$ = 1.31, *p* = 0.59; VD2: $\chi_{r}^{2}$ = 1.40, *p* = 0.53, permutation based Friedman rank sum tests).

## Discussion

In the present study, we investigated whether short-term visual deprivation modulates the ability to discriminate cross-modal emotional stimuli, that is, emotions conveyed by faces and voices. We additionally tested whether visual deprivation affects the perception of simple audio–visual (i.e., tone bursts and light flashes) stimuli as well as unimodal auditory and visual acuity, as tested with the Colavita effect and auditory and visual threshold measures, respectively. These latter tasks enabled us to further extend previous knowledge about the effects of short-term visual deprivation on basic perceptual skills (e.g., Facchini and Aglioti, 2003; Lewald, 2007; Landry et al., 2013). Whereas these previous studies examined the immediate and short-term effects of visual deprivation (i.e., the change in performance within a few hours following re-exposure to light), we adopted a longitudinal approach to document possible long-lasting effects (i.e., we tested our participants on average 8 h and 4 weeks after visual deprivation). Furthermore, we controlled for the role of visual deprivation *per se* by visually depriving an additional group of participants who concurrently performed unrelated (i.e., tactile) tasks to the later tested abilities.

Our results showed that audio–visual emotion discrimination was affected by short-term visual deprivation. In particular, we found that our visually deprived groups (VD1 and VD2) displayed enhanced affective prosodic discrimination performance in the presence of interfering incongruent affective facial expressions as a consequence of visual deprivation, whereas no significant modulation was observed in ND. Indeed, shortly after visual deprivation (session 2), VD1 and VD2 more accurately discriminated prosody in the audio–visual incongruent condition (in which a face and a voice were presented simultaneously but displayed different emotions), suggesting that visual deprivation had increased their ability to discriminate affective prosodic information in a situation of cross-modal conflict. Because only the two visually deprived groups and not the ND group showed better prosodic discrimination abilities in the context of interference from task-irrelevant facial expressions, we are able to exclude that this change may be the consequence of exposure to auditory stimulation during visual deprivation, since ND received the same set of auditory tasks as VD1 did. Previous studies did not control as thoroughly as we did for this factor (cf. Lewald, 2007; Landry et al., 2013). For example, Lewald (2007) allowed blindfolded participants to engage in conversations during visual deprivation, thus not providing conclusive results on the role of visual deprivation *per se* on modulation of perception. It has to be noted that the VD1 and VD2 groups both differed from the ND group but not from each other despite the very different tasks used during the deprivation period (auditory vs. tactile) and the unequal intervals between the end of the deprivation and the second session (the first post-deprivation session) Therefore, our results additionally reinforce the notion that visual deprivation *per se* triggers changes at the behavioral level in healthy adults.

The nature of the effect we observed needs further discussion. Notably, our visually deprived participants improved their affective prosodic discrimination abilities only in a situation of cross-modal conflict and not as a general pattern (i.e., they did not show enhanced performance in the unimodal auditory and audio–visual congruent conditions of the “attend voices” block following visual deprivation). This result could be attributed to an improved ability to segregate auditory from visual information, in particular to inhibit task irrelevant visual stimuli (improved intermodal attention). Previous studies have demonstrated that the ability to selectively attend to one modality during incongruent cross-modal stimulation may indeed be affected by alterations in sensory experience.

For example, cochlear implant (CI) users with low performance level in auditory-only speech comprehension (non-proficient CI users) were found to display an additional decrease of performance when simultaneously presented with auditory and incongruent visual speech information (i.e., lip motion). This performance decrease was significantly larger as compared to that of matched controls with similar performance in auditory-only speech comprehension (Champoux et al., 2009). In turn, non-proficient CI users were shown to display a significantly smaller decrease in visual-only speech comprehension (i.e., speech reading) in the presence of incongruent auditory speech stimuli as compared to controls (Landry et al., 2012). It could be speculated that even a short-term sensory deprivation results in a reweighting of sensory evidence in perception. Indeed, recent studies have demonstrated that emotional conditioning and reward manipulations change the weighting of sensory input when being integrated (Maiworm et al., 2012; Bruns et al., 2014). Experimental visual deprivation might, thus, change the relative weights allocated to the auditory and the visual channel.

Here the question arises of how improvement in segregating auditory from visual information in order to discriminate prosody can be reconciled with unchanged performance in discriminating affective facial expressions (i.e., the visually deprived participants did not show decreased accuracy in the unimodal visual and cross-modal incongruent conditions of the “attend faces” block following visual deprivation). We speculate that the participants’ affective facial expression discrimination performance was at a too high level to suffer from short-term visual deprivation (i.e., ceiling effect). Notably, our data showed that while affective prosodic discrimination generally declined in the context of incongruent affective facial expressions relative to unimodal stimulation (significant cross-modal interference effect across groups and sessions in the “attend voices” block) the analogous effect was not significant for affective facial expression discrimination (i.e., in the “attend faces” block). This pattern of results is in accord with previous studies (e.g., Collignon et al., 2008; Paulmann and Pell, 2011), demonstrating a dominance of the visual channel in human emotion discrimination. It is possible that while short-term visual deprivation may induce a (temporary) enhancement in the selective processing of auditory information when the concurrent visual information is task irrelevant, it might not be capable of affecting the general dominance of vision in cross-modal emotion processing.

Moreover, our results imply that short-term visual deprivation may have long-lasting effects on affective prosodic discrimination (in a cross-modal situation), since the improvement was partially still evident in the third session 4 weeks after the visual deprivation experience (i.e., there was a significant improvement in VD2 and a trend towards an improvement in VD1).

We did not observe any predicted change in visual discrimination thresholds and the Colavita effect. The differences we found (auditory discrimination thresholds for pitch were lowered in ND in session 2 and in VD2 in sessions 2 and 3, respectively) were not predicted and are hard to interpret. The largest changes in auditory perception were expected for VD1, as these participants both received an auditory training and were visually deprived. However, we did not find any change in auditory thresholds in this group while such changes were observed in the ND and VD2 groups. Taken together, our data on basic perception seem to contrast previous studies that found changes in basic non-visual abilities following short-term visual deprivation (Facchini and Aglioti, 2003; Lewald, 2007; Landry et al., 2013).

The main difference between our study and previous investigations is that we tested our visually deprived participants on average 8 h after visual deprivation. On the contrary, previous studies have tested their participants immediately after visual deprivation (Facchini and Aglioti, 2003; Lewald, 2007; Landry et al., 2013). In fact, our VD1 participants were tested between 2 and 24 h after re-exposure to light, and thus in a time window during which performance changes have been suggested to already return to baseline (Boroojerdi et al., 2000; Facchini and Aglioti, 2003; Lewald, 2007; Pitskel et al., 2007; Landry et al., 2013). However, our VD2 participants were all tested immediately after visual deprivation. Because we found a similar pattern of results in VD1 and VD2 (significant performance improvement selectively in the incongruent condition of the ‘attend voices’ block in session 2, which was still significant or showed a trend toward significance in session 3 in VD2 and VD1, respectively), the average delay in testing following visual deprivation cannot fully explain the differing results of our study and others. Therefore, our data likely exclude the possibility that time at which participants were tested following the deprivation influenced our findings. On the contrary, our results, although limited due to small sample sizes, suggest that a short period of visual deprivation may have long-term effects.

In accord with our findings of a lack of changes in basic perceptual tasks, there is at least one recent study that failed to find effects of short-term visual deprivation on basic non-visual (i.e., tactile) abilities despite larger sample sizes (29 to 32): Wong et al. (2011) assessed the performance of two groups of participants in a passive tactile GOT before and after a 110 min time interval, during which one group was visually deprived in a dark room. Results showed no effect of group or testing session, indicating that visual deprivation did not affect tactile acuity.

## Conclusion

Our study suggests that short-term visual deprivation is capable of inducing performance changes in multisensory processing. Short-term visual deprivation reduced the influence of task-irrelevant facial expressions on affective prosody judgments. Moreover, our data partially suggest a longer durability of deprivation induced effects than has previously been reported.

## Conflict of Interest Statement

The authors declare that the research was conducted in the absence of any commercial or financial relationships that could be construed as a potential conflict of interest.

## Footnotes

1 (http://www.dialog-im-dunkeln.de/en). The Dialogue in the Dark^®^ is a non-profit social enterprise with the aim of confronting sighted people with perceptual experiences in the dark while being supervised by blind guides and trainers. The person who was in charge of performing the auditory tasks for our study was a professional blind trainer.

2 The ND participants were administered the auditory tasks in the same but lit room of the Dialogue in the Dark, supervised by the same professional blind trainer who performed the tasks with VD1 as well.
